# Data Analysis and Knowledge Mining of Machine Learning in Soil Corrosion Factors of the Pipeline Safety

**DOI:** 10.1155/2022/9523878

**Published:** 2022-05-06

**Authors:** Zhifeng Zhao, Mingyuan Chen, Heng Fan, Nailu Zhang

**Affiliations:** ^1^School of Electronic Engineering, Xi'an Shiyou University, Xi'an 710065, China; ^2^Department of Mechanical, Industrial and Aerospace Engineering, Concordia University, Montreal H3G2W1, Canada

## Abstract

The purpose of this research is to enhance the ability of data analysis and knowledge mining in soil corrosion factors of the pipeline. According to its multifactor characteristics, the rough set algorithm is directly used to analyze and process the observation data without considering any prior information. We apply rough set algorithm to delete the duplicate same information and redundant items and simplify the condition attributes and decision indicators from the decision table. Combined with the simplified index, the decision tree method is used to analyze the root node and branch node of it, and the knowledge decision model is constructed. With the Python machine learning language and PyCharm Community Edition software, the algorithm functions of rough set and decision tree are realized, so as to carry out artificial intelligence analysis and judgment of the soil corrosion factor data in pipeline. Taking the area of loam soil corrosion as an example, the data analysis and knowledge mining of its multifactors original data are carried out through the model. The example verifies that the evaluation and classification rules of the model meet the requirements, and there are no problems such as inconsistency and heterogeneity. It provides decision-making service and theoretical basis for the soil corrosion management of pipeline.

## 1. Introduction

The pipeline transportation has the characteristics of high efficiency, low cost, and passing through various working conditions. It plays an irreplaceable role in energy transportation. Once the pipeline accident occurs, it will not only bring huge economic losses but also lead to casualties and environmental pollution. As the systematic mode of safety management, the pipeline integrity management is the practice embodiment of pipeline safety management for many years [1]. The pipeline integrity management is based on data collection, storage, cleaning, data analysis, and mining. Data analysis and mining in pipeline integrity management are very important. It is the basic core of integrity management and the premise of efficient application and serves the decision-making of pipeline safe transportation. Management and analysis of the soil corrosion data is an important item of the external corrosion of pipeline safety management. With the differences of pipeline working conditions and regions, the factors and sizes of multiple factors are also different, and the selection of multiple factors of soil corrosion is different too. These lead to incomplete selection of soil corrosion parameters in pipeline integrity management and failure to consider the relationship between important corrosion environmental factors (such as soil resistivity, redox potential, water content, and soil pH value) and region [2]. In this case, the data analysis is incomplete, and the results are one-sided or even wrong, which affect the correctness of pipeline integrity management decisions. At present, the main methods for the pipeline in soil corrosion factors and data analysis are as follows [3].

The single-factor index method only considers the single-factor index of soil corrosion and is one-sided.

Fault tree analysis: this method has some shortcomings in the analysis of structural importance. For example, the minimal path set or cut set method is to determine the influence of the basic events of the accident tree by Boolean algebra operation. It is simple and not accurate enough. When the minimum path set and the minimum cut set are used to analyze the same accident, the sorting results of the two kinds are inconsistent. The structural importance coefficient method needs to find out the state value relationship between basic events and top events and list them in the calculation process. It is solved by substituting the state relationship into the formula of the structural importance coefficient. The solution process is complex and cumbersome. The results are accurate relative to the minimum path set or cut set method. However, there are a certain number of basic events. For example, if the number of basic events is 8, the number of incompatible two state combinations is 2^8^ (256). Manual calculation takes a long time and is difficult to complete. The above two methods do not consider the difficulty of the basic event of the accident, and assume that the probability of the basic event leading to the accident is equal. This is inconsistent with the change process of nonequilibrium, complex, and nonlinear random variables in the actual corrosion leakage process. It can be seen that its preconditions are obviously subjective and one-sided. Probability and critical importance analysis is to calculate the relationship between the attribute size of index factors through the probability of events, and the solution steps are relatively simple. However, it is difficult to calculate the probability of top events, and when the number of basic events is too large, it is easy to produce the problem of combined storm. Although the probability of top events can sometimes be calculated according to a large number of historical statistical data, the targeted accident results are different due to regional differences [4, 5].

Principal component analysis (PCA): its eigenvalue decomposition has some limitations. For example, the transformed matrix must be a square matrix, and in the case of non-Gaussian distribution, the principal element obtained by the PCA method may not be optimal [6].

Extension analytic hierarchy process for the soil corrosion: the determination of its weight coefficient is subjective, which will greatly affect the correctness of the analysis results [7].

Multiple linear regression analysis: it requires a lot of data. In the regression analysis, which factor is selected and which expression is adopted by this factor are only a speculation. These affect the immeasurability of some factors and limit the regression analysis in some cases [8].

Failure probability analysis method: it is a statistical analysis of soil corrosion characteristics based on historical data. Using Weibull probability density distribution and other correlation functions, the probability statistical distribution of defect failure is obtained, and the parameters in the function can be changed to reflect the corrosion development characteristics and severity in different stages. However, the data based on time-series analysis method depends on historical statistics, and most of the mathematical models are simple models based on linear relationship. It is difficult for the model to accurately describe the time series of nonequilibrium, complex, and nonlinear random variable change process in the actual process. The model itself also lacks self-learning ability, and the accuracy of its analysis needs to be improved [9]. Due to the limitations of the above methods, the accuracy of prediction and prevention in pipeline integrity management are not high, and the timeliness is poor, so the due effect of integrity management is lost.

## 2. Methods

In view of the above problems, the methods of rough set and decision tree are proposed to analyze pipeline soil corrosion factors and data, combined with Python machine learning language and PyCharm community edition software.

### 2.1. Rough Set Theory (RS)

It is a mathematical tool to deal with uncertain problems. With the direct observation data, the rough set algorithm is used to delete the duplicate information and redundant items and simplify the condition attributes and the decision indicators from the decision table without considering any prior information [10].

The RS steps of data mining and weight analysis are as follows:

(a) Establish knowledge base: the actual objective data of each index attribute is used to form the information table of attribute object. A list of attributes corresponds to the equivalence relationship of an object. A table is a series of equivalence relations defined.
(b) Establish a decision table: the conditional attributes of the information table are discretized and simplified according to the decision attributes. We remove duplicate rows and error data from the information table. We simplify condition attributes to form a decision table.
(c) Attribute importance analysis (*D* is the decision attribute and *C* is the condition attribute): after checking the results of *r*_*c*_(*D*) − *r*_*c*−*i*_(*D*), we analyze their impact on decision-making attributes, delete those that have no impact, and calculate the importance of attributes that have impact.
(d) Rank *q*(*a*_*i*_) as the importance of attributes (*n* = *C* = the number of condition attribute):
  (1) $$\begin{array}{c} q\left(a_{i}\right)=\frac{r_{c}\left(D\right)-r_{c-i}\left(D\right)}{\sum_{i=1}^{n}\left[r_{c}\left(D\right)-r_{c-i}\left(D\right)\right]}. \end{array}$$

The advantage of this method is that it does not need any prior information, only excavates, analyzes, and classifies the implicit knowledge of the objective data itself. It has fault tolerance and generalization capability [11].

### 2.2. Decision Tree Analysis Method (Knowledge Decision)

Decision tree is an analysis method that can be used for knowledge decision-making. It takes the recursive classification of the tree structure as the model. It takes the data of index factors as the set space and uses the tree structure to classify the spatial attributes for decision-making. The root node is based on the requirements of index factor classification. Each subnode is a classification problem of index factors. It is classified into two or more blocks according to the level of index factors. Each block can continue to be classified until the generation of leaf nodes. A leaf node is the level classification under the condition of multiple indicator attributes. Each path from the root node to the leaf node represents a classification rule [12].

The steps of decision tree analysis (knowledge decision) are as follows:

- According to the hierarchical index factors of RS analysis, the root node and branch node of decision tree are analyzed, and attribute reduction is carried out.
- Selecting the node of decision tree: we select the core factor as the root node of the decision tree. We select branch nodes according to the weight or importance of attribute structure.
- Pruning of decision tree: the repeated classification and opposite judgment are deleted to improve the fault tolerance and adaptability of hierarchical evaluation.
- Selecting the result attribute: the corrosion grade is used as the leaf node of decision tree classification, and the evaluation model of decision tree is established [13].

### 2.3. Multifactors' Case in the Data Analysis and Knowledge Mining of Pipeline Soil Corrosion

Taking the corrosion area of loam soil as an example, the mathematical method based on rough set and decision tree are used to mine and analyze the original data of soil corrosion factors, combined with Python machine learning language and PyCharm community edition software, so that it can provide decision-making services for the management of pipelines in this area.

#### 2.3.1. Data Analysis

With the buried area and location of loam corrosion site, six influencing factors are analyzed according to the test piece data and collection batch. We used randomly selected 20 groups of corrosion data for data mining.  Table 1 shows the actual original sample of the index factor value of 20 groups' soil corrosion for the loam area section [14].

According to the rough set method, the actual sample of index factor values of soil corrosion for the loam area pipe section in Table 1 is taken as the decision table. The selected point of pipeline soil corrosion is taken as the research object, *I* = {*I*_1_, *I*_2_,…, *I*_20_}. The selected pipeline soil corrosion of influencing factors is taken as the conditional attribute, *T* = {soil resistivity, redox potential, chloride ion content, sulfuric acid root ion content, water content, pH value}. The soil corrosion grade of the pipeline in the loam area is taken as the decision attribute *J* = {average corrosion rate} = {very strong, strong, general, weak} = {4, 3, 2, 1} because the existing discrete data methods have more or less lost value problems. When the attribute value increases, the number of breakpoints will also increase. The choice of breakpoints is directly related to the correctness of discrete data. Too few breakpoints will cause serious value loss. Too many breakpoints will increase the dimension and complexity and reduce the accuracy, for example, the equal width and equal frequency interval discretization method, the statistical discretization method, the greedy and improved discretization method, the clustering continuous attribute discretization method, and the differential evolution discretization method [15, 16] This study combines the requirements and purposes of discretization. In other words, discretization should ensure the consistency and simplification of data results. Through the effectiveness of discretization, the classification ability and robustness of the dataset are improved, and the sample conflict and minimum information loss are reduced. Therefore, aiming at the discretization method and principle, it is proposed to improve its application based on the multifactor characteristics of pipeline soil corrosion, and consider the specific attribute value of the decision table (the supervised discretization method) [17, 18].  Table 1 is discretized according to its corresponding grade classification of soil corrosion. The classification of soil corrosion factors is shown in Table 2. In this way, the loss value problem in data is solved and the stability of data discretization is guaranteed. The discretization table of soil corrosion factors in the pipe section of loam area is shown in Table 3. We delete the data in brackets in data redundancy item 2 (or 10, 17), item 4 (or 7, 12), item 9 (or 15), item 11 (or 18), and item 16 (or 19, 20). The new decision table is used for attribute reduction and structural importance analysis according to the reduction decision rules.

#### 2.3.2. Attribute Reduction and Structural Importance Analysis

The importance of the condition attribute to the result attribute in the decision table can be deleted from the decision table. We calculate the size of the positive field value of the result attribute classification with removing this attribute. The influence of the attribute on the classification change of the result attribute is reflected by the size relationship of its value. The smaller the value is, the lesser the importance of the condition attribute is to the decision attribute. The larger the value is, the greater the importance of the condition attribute is to the decision attribute. Its value is zero, which means it has no impact on the result attribute and can be deleted [19].

Combined with the soil corrosion data of pipeline in loam area, the whole dataset is defined as *I*. *T* and *J* are condition attribute set and result attribute set, respectively. The condition attribute set *T* contains soil resistivity *a*, redox potential *b*, chloride ion content *c*, sulfate ion content *d*, water content *e*, and pH value *f*. The result attribute set *J* is the soil corrosion grade of loam area. That is,

(2) $$\begin{array}{c} \frac{U}{J}=\left\{\left\{2,3,8,16\right\},\left\{1,4,5,6,9,11,13,14\right\}\right\},\\ \frac{U}{T}=\left\{\left\{1,16\right\},\left\{2\right\},\left\{3,6\right\},\left\{4\right\},\left\{5\right\},\left\{8\right\},\left\{9\right\},\left\{11\right\},\left\{13\right\},\left\{14\right\}\right\},\\ \frac{U}{\left(T-a\right)}=\left\{\left\{1,16\right\},\left\{2,5\right\},\left\{3,4,6\right\},\left\{8\right\},\left\{9,13\right\},\left\{11\right\},\left\{14\right\}\right\},\\ \frac{U}{\left(T-b\right)}=\left\{\left\{1,16\right\},\left\{2,8,9\right\},\left\{3,6\right\},\left\{4,11\right\},\left\{5,13\right\},\left\{14\right\}\right\},\\ \frac{U}{\left(T-c\right)}=\left\{\left\{1,16\right\},\left\{2\right\},\left\{3,6\right\},\left\{4,14\right\},\left\{5\right\},\left\{8\right\},\left\{9\right\},\left\{11\right\},\left\{13\right\}\right\},\\ \frac{U}{\left(T-d\right)}=\left\{\left\{1,16\right\},\left\{2\right\},\left\{3,6\right\},\left\{4\right\},\left\{5\right\},\left\{8\right\},\left\{9\right\},\left\{11\right\},\left\{13\right\},\left\{14\right\}\right\},\\ \frac{U}{\left(T-e\right)}=\left\{\left\{1,2,3,6,16\right\},\left\{4,5\right\},\left\{8\right\},\left\{9\right\},\left\{11\right\},\left\{13\right\},\left\{14\right\}\right\},\\ \frac{U}{\left(T-f\right)}=\left\{\left\{1,16\right\},\left\{2\right\},\left\{3,6\right\},\left\{4\right\},\left\{5\right\},\left\{8\right\},\left\{9\right\},\left\{11\right\},\left\{13\right\},\left\{14\right\}\right\}. \end{array}$$

The positive fields of the result attributes are as follows:

(3) $$\begin{array}{c} POS_{T}\left(J\right)=\left\{2,4,5,8,9,11,13,14\right\},\\ POS_{T-a}\left(J\right)=\left\{8,9,11,13,14\right\},\\ POS_{T-b}\left(J\right)=\left\{4,5,11,13,14\right\},\\ POS_{T-c}\left(J\right)=\left\{2,4,5,8,9,11,13,14\right\},\\ POS_{T-d}\left(J\right)=\left\{2,4,5,8,9,11,13,14\right\},\\ POS_{T-e}\left(J\right)=\left\{4,5,8,9,11,13,14\right\},\\ POS_{T-f}\left(J\right)=\left\{2,4,5,8,9,11,13,14\right\}. \end{array}$$

The importance of each attribute is as follows:

(4) $$\begin{array}{c} \partial_{TJ}\left(a\right)=\frac{8}{12}-\frac{5}{12}=\frac{3}{12},\\ \partial_{TJ}\left(b\right)=\frac{8}{12}-\frac{5}{12}=\frac{3}{12},\\ \partial_{TJ}\left(c\right)=\frac{8}{12}-\frac{8}{12}=0,\\ \partial_{TJ}\left(d\right)=\frac{8}{12}-\frac{8}{12}=0,\\ \partial_{TJ}\left(e\right)=\frac{8}{12}-\frac{7}{12}=\frac{1}{12},\\ \partial_{TJ}\left(f\right)=\frac{8}{12}-\frac{8}{12}=0. \end{array}$$

**Figure 1:**
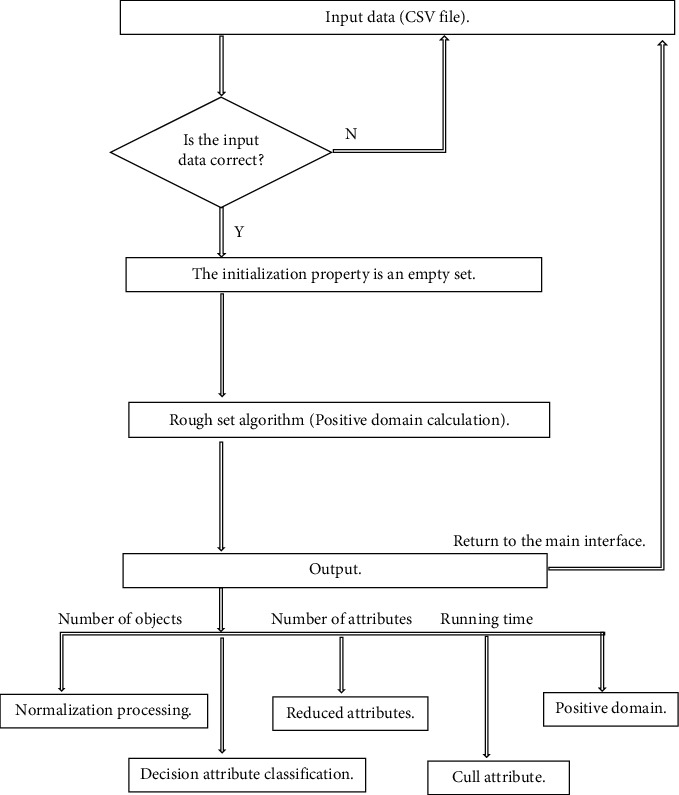
The python flowchart of rough set algorithm.

**Figure 2:**
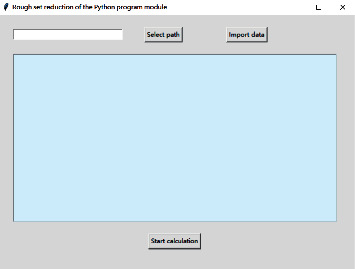
The screenshot of data import of rough set reduction in python program module.

**Figure 3:**
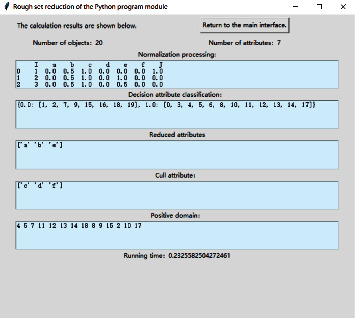
The python calculated value diagram of rough set algorithm.

We combine the application of Python machine learning language in PyCharm community edition software. Its Python program flowchart is shown in the Python flowchart of rough set algorithm in Figure 1.  Figure 2 is the screenshot of data import of rough set reduction in Python program module. The calculated results are shown in Figure 3. Figure 3 is the Python calculated value diagram of rough set algorithm [20]. The output calculation value of Figure 3 are as follows. The first item is the decision table after normalization processing. The second item is the classification item and data item under the decision attribute. The third item is the core attribute after reduction. The fourth item is the attribute that can be deleted. The fifth item is the corresponding positive field value, that is, the correct data that can be used for analysis.

According to the above calculation, the importance of influencing factors of corrosive soil pipeline in this loam area is listed as follows.

Soil resistivity = redox potential > water content > sulfate ion content = chloride ion content = pH value = 0. It indicates that the last three conditional attributes are meaningless to the results, and they can be deleted.

As can be seen from the positive field value in Figure 3, we delete duplicates of data (7 and 12, 10 and 17, and 15 and 18). The result is consistent with the above calculated value, that is, *POS*_*T*−*f*_(*J*)={2,4,5,8,9,11,13,14}, that verifies the correctness of the machine algorithm. At the same time, we delete the nonpositive field items (items 1, 3, 6, and 16) in the data, as shown in Table 4.

#### 2.3.3. Establish Decision Tree and Knowledge Mining

**Figure 4:**
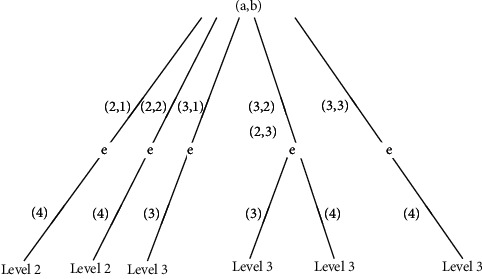
The multifactors of classification decision tree of soil corrosion in the loam area.

The key problem of establishing decision tree is the quality constructing of decision tree structure, that is, the selection of test attributes and the pruning of decision tree [21]. In order to facilitate the search for classification rules and better carry out knowledge discovery in pipeline big data, the root node of the decision tree should select the core test attributes and then construct branches through different values of the core test attributes. The branch nodes select the test attributes with large structural importance value and use the recursive classification method to establish them repeatedly. Because the characteristics of the set space of pipeline soil corrosion data will lead to the problem of overfitting, it is necessary to prune the decision tree. Therefore, it is necessary to delete the redundant items of the opposite classification rules and the repeated classification rules, so as to improve the ability of rule information classification of decision tree. It can be seen from the reduction item of pipeline soil corrosion in loam area in Table 4 that the data item 4 is repeated with item 14, and item 14 will be deleted. The attribute selection, pruning, and knowledge classification decision of decision tree are carried out by using reduction items. That is, the root node of the decision tree, the core index factors of soil resistivity, and redox potential are selected. The branch node selects the water content according to the importance of the attribute structure. The leaf node is the soil corrosion grade of the pipeline in the loam area, as shown in the multifactors of classification decision tree of soil corrosion in the loam area pipeline section, in Figure 4 [22].

### 2.4. Pipeline Section

**Figure 5:**
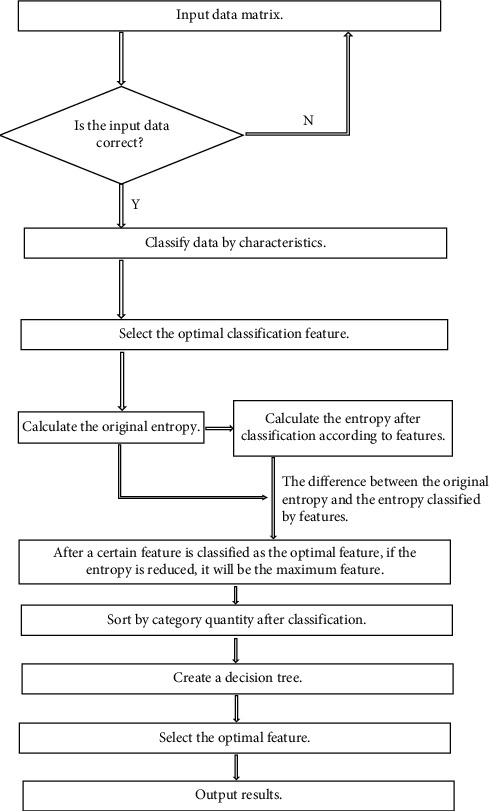
The Python flowchart of decision tree algorithm.

**Figure 6:**
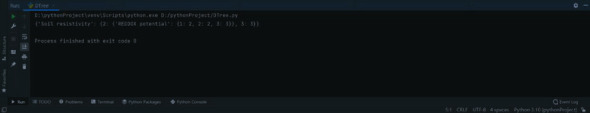
The Python calculated value diagram of decision tree algorithm.

We combine the application of Python machine learning language in PyCharm community edition software. Its Python program flowchart is shown in Figure 5. Figure 5 is the Python flowchart of decision tree algorithm. The calculated results are shown in the Python calculated value diagram of decision tree algorithm in Figure 6 [23].

According to the analysis rules in Figure 6, when the soil resistivity is grade 2, if the redox potential is grade 1 or grade 2, the soil corrosion grade is grade 2. If the redox potential is grade 3, the soil corrosion grade is grade 3. According to the analysis rules in Figure 4, when the soil resistivity and redox potential indexes are in the right range of (3, 1) index rules, the soil corrosion grade is grade 3. When the soil resistivity and redox potential index are in the left range of (3, 1) index rule, the soil corrosion grade is grade 2. In terms of (2, 3) index rule, according to the analysis of importance, it is the same as (3, 2) index rule, so it is also on the right of (3, 1) index rule, and its soil corrosion grade is also grade 3. Therefore, it can be seen that the analysis rule in Figure 4 is consistent with the analysis rule in Figure 6.

Combined with the previous analysis, from the calculation results, the order of importance is soil resistivity = redox potential > water content. As shown in Figure 6, as long as the soil resistivity or redox potential is grade 3, the soil corrosion grade is grade 3. The validation data in Table 5 also prove its consistency.

## 3. Results

The six groups of soil corrosion data measured in the loam area pipeline section are used as the inspection data. Table 5 is the inspection table of six groups of soil corrosion data measured in the loam area. According to the analysis rules in Figure 6, in Item 1 of the serial number in Table 5, if the soil resistivity is grade 2 and the redox potential is grade 1, the soil corrosion grade is judged to be grade 2 according to the results, which is consistent with the average soil corrosion rate of grade 2. In Item 4 of the serial number, if the soil resistivity is grade 2 and the redox potential is grade 2, the soil corrosion grade is judged as grade 2 according to the rules, which is consistent with the average soil corrosion rate. In Items 2, 3, 5, and 6 of the serial number, if the soil resistivity is grade 3, the soil corrosion grade is judged as grade 3 according to the rules, which is consistent with the average soil corrosion rate of grade 3. According to the results of decision tree analysis, the rule accuracy is 100%.

## 4. Discussion

Taking the average corrosion rate of soil as the decision attribute, the corrosion capacity of different soils can be objectively reflected, which meets the actual requirements. However, due to the complexity and accuracy requirements of its measurement, it is often time-consuming, which is not conducive to the field practical application. Therefore, the rough set method is used to analyze the relevant weight and importance of the actual and objective detection data of soil corrosion. The application method of discretization is improved. The classification of soil corrosion grade is used to discretize the data, so as to avoid the value loss problem and increase the applicability and objectivity of the analysis of its factors and data. The classification rules are established according to the core index factors of soil corrosion. According to the importance of multi-index factors, the root node, branch node, and leaf node in the decision tree are selected and the structure is optimized. It can visually analyze the corrosion grade of soil, so as to provide knowledge decision-making and data basis for soil corrosion analysis.

## 5. Conclusions

Based on the rough set and decision tree method, the PyCharm community edition software is used to analyze the case of pipeline soil corrosion data. With data analysis and knowledge mining, the results show that the pertinence and adaptability of pipeline integrity management can be improved only by comprehensively considering the characteristics of pipeline data and the different characteristics of the influence of environmental factors in different regions.

The importance analysis of attribute structure using the rough set method is a multivalued and nonnumerical importance processing method, which makes full use of the objective information of the original data without any prior conditions and additional information. The traditional method of attribute structure importance analysis can only deal with the problem of the binary numerical model. By using the core attributes of the rough set and the importance value of attribute structure, we can build an intuitive decision tree with easy discovery of knowledge rules, which reduces the complexity of the tree and improves the fault tolerance and classification effect.

From the model established by the decision tree based on the data reduction rules of rough set analysis, the evaluation classification rules meet the requirements, and there are no problems such as inconsistency and heterogeneity. These provide knowledge and decision-making basis for the multifactors' classification of soil corrosion in the pipeline section.

In the future, we will increase the data points, expand the amount of data collection, and carry out method training according to different soil environments through resource integration. We will find the core factors of each soil environment through the model, extract the identification feature attributes, and build a knowledge base to provide guarantee for the intelligent identification application of subsequent models and autonomous learning.

## Figures and Tables

**Table 1:** The actual original sample of index factor value of 20 groups' soil corrosion for the loam area section.

Serial number	Soil resistivity (Ω · *m*)	Reddox potential (mV)	Chloride ion (%)	Sulfuric acid root ion content (%)	Water content (%)	pH value	Average corrosion rate (g · dm^−1^ · a^−1^)	Soil corrosion grade
1	62.9425	313.533	0.0144	0.01756	8.25	7.34	5.2247	Strong
2	155.7157	302.052	0.0124	0.01534	12.45	7.44	2.8392	General
3	84.9372	338.027	0.0125	0.03491	34.37	7.55	3.8477	General
4	37.6574	306.648	0.0144	0.0376	34.12	6.89	6.2733	Strong
5	33.0159	385.396	0.0144	0.02112	12.35	7.14	6.8461	Strong
6	63.3307	311.137	0.0173	0.01154	9.33	7.16	4.7189	Strong
7	25.6742	419.935	0.0143	0.05174	8.87	7.36	6.3126	Strong
8	113.7052	502.084	0.01760	0.03651	21.96	7.23	3.2279	General
9	77.6334	167.152	0.0153	0.02493	16.95	6.43	6.2759	Strong
10	154.0341	458.893	0.0115	0.03644	20.73	7.51	2.9502	General
11	14.2288	513.172	0.0147	0.03457	26.55	7.21	6.9759	Strong
12	56.3997	421.915	0.0118	0.03455	8.77	6.73	4.3627	Strong
13	36.443	157.121	0.0142	0.03071	14.85	7.05	5.9397	Strong
14	28.2099	488.604	0.0098	0.04001	33.91	7.19	6.0967	Strong
15	102.0471	167.031	0.0171	0.04990	15.58	6.49	5.9957	Strong
16	108.9347	450.937	0.0124	0.02306	7.44	7.31	3.2401	General
17	167.3979	376.081	0.0171	0.02117	24.49	7.29	2.0774	General
18	37.1103	624.478	0.0124	0.02493	10.13	6.52	5.3439	Strong
19	136.0466	473.387	0.0124	0.02675	37.11	7.68	3.3369	General
20	71.0341	455.946	0.0150	0.01731	7.46	7.44	2.7917	General

**Table 2:** The classification interval table of the soil corrosion index factors.

Classification model	Soil resistivity (Ω · *m*)	Reddox potential (mV)	Chloride ion content (%)	Sulfuric acid root ion content (%)	Water content (%)	pH value	Average corrosion rate (g · dm^−1^ · a^−1^)
*J*1	>300	>500	<0.01	<0.009	<3	>8.5	<1
*J*2	[60, 300]	[300, 500]	[0.01, 0.075]	[0.009, 0.06]	[3, 8.5) and >35	[6.25, 8.5]	[1, 4]
*J*3	[5, 60)	[100, 300)	[0.075, 0.75)	(0.06, 0.65]	[8.5, 12) and [25, 35)	[4.5, 6.25)	(4, 7]
*J*4	<5	<100	>0.75	>0.65	[12, 25)	<4.5	>7

**Table 3:** The discretization table of soil corrosion factors in the pipe section of loam area.

Serial number	Soil resistivity (Ω · *m*)	Reddox potential (mV)	Chloride ion content (%)	Sulfuric acid root ion content (%)	Water content (%)	pH value	Soil corrosion grade
1	2	2	2	2	2	2	3
2	2	2	2	2	4	2	2
3	2	2	2	2	3	2	2
4	3	2	2	2	3	2	3
5	3	2	2	2	4	2	3
6	2	2	2	2	3	2	3
7	3	2	2	2	3	2	3
8	2	1	2	2	4	2	2
9	2	3	2	2	4	2	3
10	2	2	2	2	4	2	2
11	3	1	2	2	3	2	3
12	3	2	2	2	3	2	3
13	3	3	2	2	4	2	3
14	3	2	1	2	3	2	3
15	2	3	2	2	4	2	3
16	2	2	2	2	2	2	2
17	2	2	2	2	4	2	2
18	3	1	2	2	3	2	3
19	2	2	2	2	2	2	2
20	2	2	2	2	2	2	2

**Table 4:** The RS reduction table of the loam area of soil corrosion.

Serial number	Soil resistivity (Ω · m)	Reddox potential (mV)	Water content (%)	Soil corrosion grade
2	2	2	4	2
4	3	2	3	3
5	3	2	4	3
8	2	1	4	2
9	2	3	4	3
11	3	1	3	3
13	3	3	4	3
14	3	2	3	3

**Table 5:** The inspection table of six groups of soil corrosion data measured in the loam area.

Serial number	Soil resistivity (Ω · *m*)	Reddox potential (mV)	Water content (%)	Average corrosion rate (g · dm^−1^ · a^−1^)
1	255.7157 ⟶ level 2	502.173 ⟶ level 1	8.91 ⟶ level 3	1.9358 ⟶ level 2
2	36.4728 ⟶ level 3	310.531 ⟶ level 2	33.84 ⟶ level 3	5.4695 ⟶ level 3
3	25.7315 ⟶ level 3	157.144 ⟶ level 3	10.31 ⟶ level 3	6.7316 ⟶ level 3
4	112.4932 ⟶ level 2	422.065 ⟶ level 2	2.27 ⟶ level 1	3.4695 ⟶ level 2
5	28.3096 ⟶ level 3	501.742 ⟶ level 1	7.46 ⟶ level 2	4.1014 ⟶ level 3
6	34.7758 ⟶ level 3	306.683 ⟶ level 2	20.76 ⟶ level 4	6.1137 ⟶ level 3

## Data Availability

The raw data required for these findings cannot be shared at this time as the data also form part of an ongoing study.
